# *iCare* – a self-directed, interactive online program to improve health and wellbeing for people living with upper gastrointestinal or hepato-pancreato-biliary cancers, and their informal carers: the study protocol for a Phase II randomised controlled trial

**DOI:** 10.1186/s12885-024-11861-2

**Published:** 2024-01-29

**Authors:** Patricia M Livingston, Natalie Winter, Anna Ugalde, Liliana Orellana, Antonina Mikocka-Walus, Michael Jefford, John Zalcberg, Neil Orford, Alison M Hutchinson, Andrew Barbour, Nicole Kiss, Bernard Mark Smithers, David I Watson, Nikki McCaffrey, Victoria White, the iCare Advisory Group, Kon Mouzakis, Catherine Mihalopoulos, Katherine Lane, David W. Austin, Eric O, Kathryn Whitfield, David Menzies, Amy Larsen, Dayna Swiatek, Eva Yuen, Elysia Greenhill, Lahiru Russell, Keon Stevenson, Toni Musat

**Affiliations:** 1 Deakin University, Geelong, VIC 3220 Australia; 2 Faculty of Health, Deakin University, Geelong, VIC Australia; 3 School of Nursing &, Midwifery Deakin University, Geelong, VIC Australia; 4 Biostatistics Unit, Faculty of Health, Deakin University, Geelong, VIC Australia; 5 School of Psychology, Faculty of Health, Deakin University, Geelong, VIC Australia; 6 Department of Health Services Research, Peter MacCallum Cancer Centre, Melbourne, VIC Australia; 7 Sir Peter MacCallum Department of Oncology, University of Melbourne, Parkville, VIC Australia; 8 Department of Medical Oncology, Alfred Health and School of Public Health, Faculty of Medicine, Monash University, Melbourne, Australia; 9 Monash University, Melbourne, Australia; 10 Barwon Health, Geelong, VIC Australia; 11 Australia and New Zealand Intensive Care Research Centre (ANZICS-RC), SPHPM, Monash University, Melbourne, Australia; 12 Upper GI Unit, Princess Alexandra Hospital, Faculty of Medicine, University of Queensland, Brisbane, Australia; 13 Institute for Physical Activity & Nutrition, Faculty of Health, Deakin University, Geelong, VIC Australia; 14 Discipline of Surgery, College of Medicine and Public Health, Flinders University, Adelaide, SA Australia; 15 Deakin Health Economics, School of Health and Social Development, Faculty of Health, Deakin University, Geelong, VIC Australia

**Keywords:** Upper GI cancers, Economics of cancer, Supportive care, Web-based platforms, Online, Pancreas, Liver, Stomach, Bile duct, Oesophagus, Phase II randomised controlled trial

## Abstract

**Background:**

Up to 70% of people diagnosed with upper gastrointestinal (GI) tract or hepato-pancreato-biliary (HPB) cancers experience substantial reductions in quality of life (QoL), including high distress levels, pain, fatigue, sleep disturbances, weight loss and difficulty swallowing. With few advocacy groups and support systems for adults with upper GI or HPB cancers (i.e. pancreas, liver, stomach, bile duct and oesophageal) and their carers, online supportive care programs may represent an alternate cost-effective mechanism to support this patient group and carers. *iCare* is a self-directed, interactive, online program that provides information, resources, and psychological packages to patients and their carers from the treatment phase of their condition. The inception and development of *iCare* has been driven by consumers, advocacy groups, government and health professionals. The aims of this study are to determine the feasibility and acceptability of *iCare,* examine preliminary efficacy on health-related QoL and carer burden at 3- and 6-months post enrolment, and the potential cost-effectiveness of *iCare,* from health and societal perspectives, for both patients and carers.

**Methods and analysis:**

A Phase II randomised controlled trial. Overall, 162 people with newly diagnosed upper GI or HPB cancers and 162 carers will be recruited via the Upper GI Cancer Registry, online advertisements, or hospital clinics. Patients and carers will be randomly allocated (1:1) to the *iCare* program or usual care. Participant assessments will be at enrolment, 3- and 6-months later. The primary outcomes are i) feasibility, measured by eligibility, recruitment, response and attrition rates, and ii) acceptability, measured by engagement with *iCare* (frequency of logins, time spent using *iCare*, and use of features over the intervention period). Secondary outcomes are patient changes in QoL and unmet needs, and carer burden, unmet needs and QoL. Linear mixed models will be fitted to obtain preliminary estimates of efficacy and variability for secondary outcomes. The economic analysis will include a cost-consequences analysis where all outcomes will be compared with costs.

**Discussion:**

*iCare* provides a potential model of supportive care to improve QoL, unmet needs and burden of disease among people living with upper GI or HPB cancers and their carers.

**Australian and New Zealand Clinical Trials Registry:**

ACTRN12623001185651. This protocol reflects Version #1 26 April 2023.

## Background

Managing cancers is a significant public health and economic challenge. Healthcare costs in Australia directly attributable to cancer were $10.1Billion Australian dollars in 2015–16 [1]. There is also a growing body of evidence indicating timely access to supportive care, such as targeted information and support, can lead to improvements in quality of life and survival, as well as being cost-effective [2]. However, people living with upper gastrointestinal (GI) cancers and hepatic-pancreas-biliary cancers, in contrast to cancers with a higher public profile (i.e. breast and prostate), receive minimal support services [3].

Upper gastrointestinal (GI) cancers affect the upper digestive system and include the stomach and oesophagus. Hepato-pancreato-biliary cancers (HPB) affect the liver, pancreas and bile duct. Most recent data from the Australian Institute of Health and Welfare (AIHW) suggests the incidence of upper GI and HPB cancers in Australia is on the rise [4], with nearly 30,000 Australians diagnosed with one of these cancers each year, representing 555 new diagnoses each week [4]. Survival for upper GI and HPB cancers is low compared to more common cancers, with 5-year survival rates ranging from less than 10% to 40% depending on the cancer type [5]. In addition, up to 70% of people with these cancers experience substantial reductions in quality of life, with many living with high distress levels, ongoing pain, fatigue, sleep disturbances, unintentional weight loss and difficulty swallowing [6–8]. Interventions that can improve the QoL of those living with upper GI or HPB cancers are needed [9].

Informal carers of people living with cancer are an important component of the care team, playing a significant role in enabling patients to manage their disease, treatment and side effects outside of clinical settings [10, 11]. They are often a friend or family member who are differentiated from formal, professional caregivers within the healthcare team [12].

Previous research has highlighted the demands of caring for a person with cancer. This may be so overwhelming that providing care erodes the physical and psychological health of the carer [13]. In Australia, about 60% of the lifetime financial cost of cancer is borne by informal carers [14]. Despite the extensive involvement of informal carers in the management of people with cancer, there are limited support services for carers of people living with cancer in general, with few, if any, evidence-based solutions available to support carers of people with cancer [15, 16]. Recent studies have identified the need for more work to test interventions to support carers of people living with less common cancers [17] with interventions designed to improve mental health particularly needed for carers of people with advanced cancer [18].

Technology-based approaches can be specifically designed to bring evidence-based information from diverse sources and can provide tailored information and support for people affected by cancer [19, 20]. Such approaches can empower people with cancer and their carers, and provide a strong support tool for patients with complex health conditions [21]. Web-based interactive portals can be a cost-effective and user-centred strategy to provide support over the long term, as information and supportive care strategies can be accessed when they are needed rather than when a health professional is available. There is a strong imperative to determine whether web-based portals are effective and cost-effective in improving health outcomes for people affected by complex health conditions, such as those living with upper GI or HPB cancers.

Previous studies have identified that education, information, and tracking systems are needed to promote self-management for people with gastric cancers [22]. To our knowledge there are no current digital health interventions that incorporate information, online psychological programs, and access to community resources to support people diagnosed with upper GI and HPB cancers and those caring for them.

*iCare* has been developed to address the gap in support for people with upper GI and HPB cancers. The content and structure of the program were developed through consultation with our partner organisations and consumers and include links to information, resources, and access to psychological programs. The portal was pilot tested involving a sample of 10 patients and carers [23]. All participants reported *iCare* was acceptable and useable, with comments such as ‘*this is very valuable; there’s a lack of information and resources available’*; ‘*the portal is easy to use;’ ‘good for carers to feel supported too’;* ‘*a platform like iCare that provides ongoing support would be great’; ‘the carer is a huge part of the patient’s journey and it is important to include carers with this platform’, and ‘it’s important to advocate’.*

## Methods and analysis

### Primary aim

1. To determine the feasibility and acceptability of *iCare* for people living with upper GI or HPB cancers, and their carers. Feasibility, i.e. eligibility, recruitment, response and attrition rates; and acceptability, i.e., engagement with *iCare*, including frequency of logins, time spent use of the program and different features over the intervention period.

### Secondary aims

2. To examine the preliminary efficacy of *iCare* on people living with upper GI and HPB cancers QoL, using the Functional Assessment of Cancer Therapy – General (FACT-G) Scale; [24] and unmet needs, using the Supportive Care Needs Survey (SCNS) [25], at 3- and 6-months post enrolment;

3. To examine preliminary efficacy of *iCare* on carer burden, using the Zarit Burden Short Form 12 (ZBI-12) scale [26], unmet needs, using the Partner and Caregiver Supportive Care Needs Survey (SCNS-P&C) [27]; and QoL, using the Caregiver Quality of Life Index-Cancer (CQOLC) scale [28], at 3- and 6-months post enrolment;
4. To explore the potential cost-effectiveness of *iCare* from health and societal perspectives, at 3- and 6-months post enrolment, for both patients and carers;
5. To undertake a process evaluation, guided by the RE-AIM Framework [29], conducted in parallel with the RCT.

### Study design

**Fig. 1:**
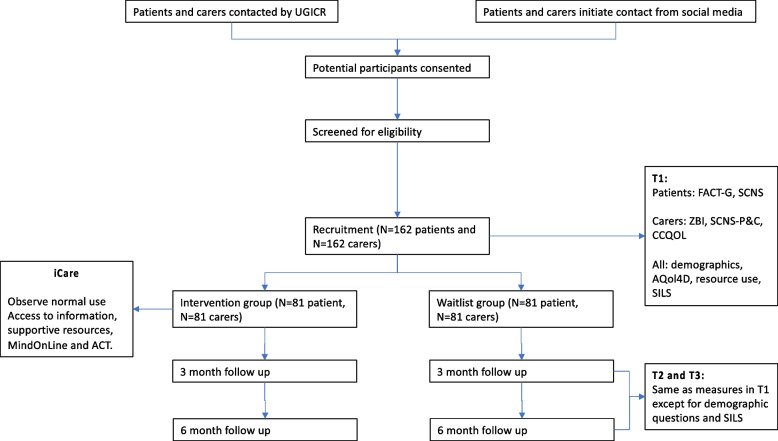
Schedule of questionnaires * Functional Assessment of Cancer Therapy (FACT-G); Supportive Care Needs Survey (SCNS); Zarit Burden Interview-12 (ZBI-12); Partner and Caregiver Supportive Care Needs Survey (SCNS-P&C); Caregiver Quality of Life Index-Cancer (CQOLC); AQoL-4D (Assessment of Quality of Life – 4 Dimensions); SILS (Single Item Literacy Scale), Upper Gastrointestinal Cancer Registry (UGICR)

The design is an Australian based phase II randomised controlled trial (RCT) where people diagnosed with upper GI or HPB cancers and their carers will be recruited and allocated (1:1) to receive *iCare* or usual care during a 3-month period. The time period recognises patients’ complex physiological and psychological needs and carer support needs, with outcomes, measured at enrolment (Time 1), 3 months (Time 2) and 6 months post-enrolment (Time 3, Fig. 1).

### Selection criteria

Patients will be eligible if they are:

- aged 18 years or older
- diagnosed with upper GI or HPB cancers in the previous 4 months
- expected minimum survival of 6 months
- able to speak and read English
- access to a web-based device, such as a smartphone, tablet, laptop, desktop computer or other internet connected device.

Carers will be eligible if they:

- identify as a person involved in the day-to-day care and medical appointments of someone with upper GI or HPB cancer
- are aged 18 years or older and
- have access to a web-based device, such as a smartphone, tablet, laptop, desktop computer or other internet connected device.

### Setting

#### Upper gastrointestinal cancer registry

Participants will be recruited primarily from the Upper Gastrointestinal Cancer Registry (UGICR) [30]. The UGICR is a clinical quality registry that receives an average of 830 reported Upper GI and HPB cancer cases each year. The Registry receives notifications of people diagnosed with these cancers from state-based cancer registries, individual hospital or surgical centres, pathology providers, and private clinicians’ rooms and after contacting people to allow them to opt out of the registry, the registry collects information on the diagnosis, treatment and outcomes of individuals with these cancers with a view to improve patient outcomes and quality of care. In general, people with our eligible cancers are registered with the UGICR within 3 months of diagnosis. 

People registered with the UGICR will be screened for study eligibility by registry staff, with those eligible sent a letter from the UGICR inviting them to participate in the trial. The invitation letter will also invite carers into the study. We aim to recruit patient-carer dyads, however, if one member of the dyad chooses not to participate, the other will still be eligible to participate in the trial.

Interested individuals (patients and/or carers) will use a Uniform Resource Locator (URL) or Quick Response (QR) code to access the study website to obtain more information, to register and to complete the first survey. UGICR letters will also include the contact details of the project manager and potential participants can email or call for further information.

If no response is received within 2 weeks, a follow-up invitation phone call will be made by registry staff to people who met the eligibility criteria.

#### Social media and clinical recruitment

Recruitment will be monitored weekly; if UGICR recruitment does not generate sufficient participation levels within 18 months, recruitment of patients and carers will be supplemented by:

i. social media: advertisements via Meta (Facebook and Instagram) will include a brief description of the study e.g., ‘If you would like to try a web-program for people living with upper GI cancer or their carers, click here’. A link on the Meta advertisement will direct those interested to the iCare homepage for full study information; and
ii. participating health services’ oncologists or surgeons involved in the project. The clinician at participating sites will screen patients at outpatient clinical appointments and provide patients and carers with a flyer highlighting the details of the study and contact information of the project manager.

### Consent and screening

Once directed to the *iCare* website, participants will be invited to register by entering their name and email address. Following this they will be presented with the plain language statement for reading and then asked if they consent. Participants will have the option to invite either their carer (main support person) or the person living with cancer to join the study. Participants who would like to invite someone to join the study will be asked to provide the name, email address and phone number of the invitee and an automatically generated email invitation will be sent to them. Phone numbers are collected as a quality control measure to link participants together.

Potential participants will complete an online eligibility survey screening for age, type of cancer, and time since diagnosis. Eligible participants will receive an on-screen message informing them they are eligible. People who are not eligible will receive a message on screen thanking them for their interest in the study and referring them to the Cancer Council 13 11 20 telephone service [31] if they require support. Those eligible will be directed to the enrolment questionnaire via Qualtrics. After completing the enrolment questionnaire participants, carers or patients, will receive a follow-up email confirming their group allocation.

### Randomisation

The first member of the patient-carer dyad will be randomly assigned (1:1 ratio) to intervention or usual care (stratified by cancer type). If the 2nd member of the dyad consents, they will be allocated to the same arm as the other dyad member. To ensure that dyads are accurately allocated to the same arm, an embedded system within iCare will match email addresses and phone numbers provided during registration.

Consenting participants who do not sign up as part of a dyad (i.e., patients, carers or the patient-carer dyad) will be randomly assigned (1:1 ratio) to intervention or usual care (stratified by cancer type). The random sequence will be embedded in Qualtrics, ensuring allocation concealment. The biostatistician, co-investigators and associate investigators will be blinded to group allocation.

#### Usual care group 

Participants allocated to the usual care arm (patients and carers) will receive at enrolment, information about support, including recommendations to use services, such as Pancare Foundation and the Cancer Council telephone support line (both staffed by cancer nurses). At the end of the data collection period (6-months post enrolment), participants in the usual care arm will be offered access to *iCare* for their ongoing use.

#### Intervention group

Participants (patients and carers) allocated to the intervention arm will be automatically redirected to *iCare*’s main menu page after completing the enrolment questionnaire. This will allow for seamless entry into the program. Participants will also be sent an email with a link to the *iCare* program, a user identification number and instructions for how to download the program to their preferred device (ie, smartphone, tablet, laptop etc.) for future use. Video instructions on how to use *iCare* will be provided. Automatic email notifications will be sent weekly highlighting information and resources within *iCare*. Participants will be able to access *iCare* as needed (i.e., no frequency/module release/duration limits), allowing for observation of natural use. Participants will have continued access to *iCare* following completion of the study.

### *iCare* program content

*iCare* is a website developed by Research Technology Architects at Deakin University in collaboration with our co-investigators, partners and consumers. Consumer / stakeholder input, research evidence, guidelines and recommendations informed the modules on *iCare* which provide resources for patients and carers. The program comprises two system pathways (patient or carer). The relevant pathway will be identified at login stage. Similar to our previous work [32, 33], the pathways will provide information, support and resources with an additional facility to identify and record pain for each cancer type.

*iCare* is an interactive platform, organised around 15 modules, with versions for people diagnosed with different upper GI or HPB (pancreas, liver, stomach, bile ducts, oesophagus) cancers.

**Fig. 2:**
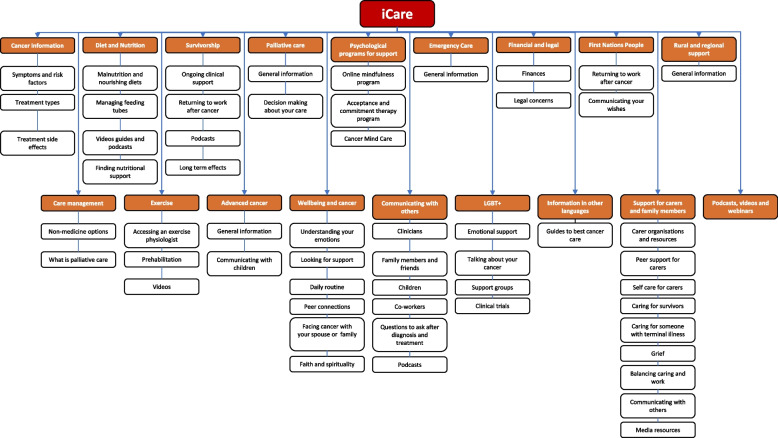
Each landing page for the different cancer types includes 18 modules

*iCare* provides relevant information in each module through a mix of video, podcasts and written resources (Fig. 2). Each time a patient accesses the program, they provide information on their current physical and emotional needs and an algorithm directs them to relevant information on the website with all information on the website still able to be accessed. The program is fully flexible allowing participants to engage in the modules most relevant to their needs over the 3-month intervention period.

### Data collection

Information will be collected at enrolment (T1), 3 (T2) and 6 months (T3) post enrolment. All patient and carer participants will be emailed a link to the online questionnaires administered through Qualtrics at T1, T2 and T3. Each questionnaire will take approximately 20 min to complete. Participants who do not complete questionnaires within 2 weeks will be followed up by telephone at each data collection point. Particpants will receive 2 reminder telephone calls, surveys not returned within 4 weeks after each data collection point will be marked as missing.

Demographic data for intervention and usual care groups: (collected at enrolment T1): age, gender, postcode, date of diagnosis, treatment, length of time in the caring role, patient diagnosis and patient treatment (carer), living situation, distance to treating health service, highest level of education, identification with lesbian, gay, bisexual, transgender, queer, intersex, asexual communities and identification as First Nations or Aboriginal and/or Torres Strait Islander people. Both patient and carer groups will complete a Single Item Literacy Screener (SILS) question that identifies whether participants need assistance to read and understand health information [34]. Health literacy will allow us to assess the health literacy levels of study participants and explore the relationship between health literacy and website use.

#### Feasibility and acceptability of *iCare* (Aim 1)

Feasibility will be measured by: eligibility, recruitment, and retention rates over the 6-month study period. Acceptability will be measured by usage data (e.g. usage patterns, number and length of log-ins, pages viewed, use of interactive features including symptom tracking calendars, the number of external websites accessed via links in the program and number of days between first and last log-in), iCare usage [35] for both patients and carers. Data tracking is embedded within *iCare;* this will be summarised to determine engagement. Satisfaction with the program will also be collected from open ended questions (T2) for the intervention group.

#### Patient outcomes (Aim 2)

*Functional Assessment of Cancer Therapy (FACT-G)* [24] is a 27-item questionnaire, designed to measure four domains of quality of life in people living with cancer and valid for all cancer types.

*Supportive Care Needs Survey (SCNS)* [25] is a 34-item instrument that comprises five main themes (psychologic, health system and information, physical and daily living, patient care and support, and sexuality). It will provide information on the potential impact of *iCare* on patient unmet needs.

#### Carer outcomes (Aim 3)

*Zarit Burden Interview-12 (ZBI-12)scale* [26], a short version of the standard ZBI (22 questions), is a reliable and valid measure of carer burden. The ZBI has been used across a variety of cancer types as well as across different stages of disease.

*Partner and Caregiver Supportive Care Needs Survey (SCNS-P&C)* [27] comprises 45 items that can determine carers' unmet needs, prioritise health-care resources, and tailor supportive cancer care services. It includes subscales of psychological, emotional, work, social and information.

*Caregiver Quality of Life Index-Cancer (CQOLC) scale* [28] measures quality of life and provides information about physical, social, emotional and financial wellbeing. It comprises 35 questions, and has been used in technology-based intervention trials [36].

#### Cost effectiveness data (Aim 4)

The Assessment of Quality of Life – four Dimensions (AQoL4D) [37] will be used to capture health-related quality of life scores and calculate quality-adjusted life years (QALYs) for both patient and carers. QALYs are the preferred outcome metric of economic evaluations as they allow practical ‘value-for-money’ judgements to be made. Program use will be recorded over the intervention period and beyond, via both automated website analytics and self-report satisfaction at T2.

Resource use and costs will be measured via self-report. A questionnaire, previously used by our team in other cancer-related studies, will be used to capture patients’ use of general medical resources, including hospital use (emergency department and admissions) welfare, and productivity losses [38].

### Program satisfaction

Participants in the intervention group will be asked to provide feedback about *iCare*. Quantitative and qualitative data will be collected, including open ended questions on participant satisfaction with the program content, uptake of the program, usability, acceptability, and areas for improvement.

### Sample size and power calculation

This Phase II RCT will provide insights into the intervention feasibility and acceptability as well as estimates of efficacy of the iCare intervention on a range of measures to inform decisions about a larger Phase III trial. We plan to recruit a minimum of 162 patients and 162 carers as dyads or as independent participants. We have successfully recruited patients and carers in the past [39]. An average of 830 Upper GI and HPB cancer cases are reported to the UGICR every year (personal correspondence; September 2023). Assuming, on average, 38% (personal correspondence, UGICR; September 2023) will be eligible and assuming a conservative 26% [39] recruitment rate, we expect to recruit ~ 82 patients and carers per year or 164 in each cohort over 24 months. This pragmatic sample size will provide adequate information to report recruitment and attrition rates, study feasibility and acceptability outcomes for both patients and carers. The sample size will allow preliminary estimation of the treatment effects and between and within participant variability for both patient and carer outcomes to inform Phase III trial planning [40]. Assuming 33% attrition at 3 months [25, 39], complete data from 108 patients and 108 carers (54 per arm) would achieve 80% power to detect large standardised mean differences (Cohen’s d) of 0.54. For the main patient outcome, FACT-G [24] these effect sizes correspond to a mean difference of 13 points between groups (standard deviation = 24) [24]. Clinically Meaningful Changes for the FACT-G scale are in the range of 5–6 [40].

### Analysis plan

Results will be presented for patients and carers independently.

#### Aim 1

We will report on recruitment, adherence, retention, accrual, attrition rates, monthly enrolment by upper GI and HPB cancer type (pancreas, liver, stomach, bile ducts and oesophageal) and characteristics of sample, and determination of productive recruitment methods, and survey completion rates. Reasons for attrition will be ascertained from participants/carers and the MORECare Guidelines will be applied to report and manage attrition [41]. We will report measures of engagement and explore, whether usage of the web-based program is associated with better outcomes in both patients and carers after controlling for potential confounders. Responses to the satisfaction questionnaire (open-ended questions) will be analysed using thematic analysis to identify themes and subthemes in the data.

#### Aims 2–3

Preliminary effect size and variance estimates for patient (QoL, unmet needs) and carer (burden, unmet needs, QoL) outcomes will be estimated using linear mixed models. The models will include study arm (*iCare/*usual care), time (T1, T2, T3) and time by arm interaction as fixed effects and patient (or carer) as random effect. Models will include the randomisation stratification factor (cancer type) as a fixed effect. Estimates of effect, variability, and intraclass correlation coefficients will be reported for each outcome alongside 95% confidence intervals.

#### Economic Evaluation (Aim 4)

A cost-consequences analysis will explore the potential incremental costs and the full spectrum of outcomes via a series of cost-effectiveness ratios. The AQoL4D will allow a cost-utility examination to be undertaken (cost/QALY). Intervention and other costs will be estimated from the study financial records (including consideration of *iCare* development costs), self-report hospital data and the Resource Use Questionnaire (including health care, welfare, and employment (unpaid work). Standardised economic evaluation techniques, such as incremental analysis of mean differences, generalised linear modelling techniques and bootstrapping to determine confidence intervals will be used. The AqoL4D will also be applied to determine economic and social benefits for carers.

#### Process evaluation (Aim 5)

The RE-AIM Framework will guide an evaluation of the uptake of *iCare*. Following RE-AIM, data captured through the RCT will assess: Reach [R] – Demographic characteristics of participants will be captured to enable a description and comparison of the sample as a sub-set of the target population. Effectiveness [E] assessed on QoL, unmet needs, carer burden and cost-effectiveness through the RCT. Adoption [A] – from RCT: use of *iCare* over the 3-month intervention period. Surveys will be conducted with participants regarding acceptability and feasibility of *iCare*; perceived barriers and facilitators of use; and the degree to which *iCare* addressed their needs. Google Analytics data will evaluate participants’ *iCare* activity, including login date/times, navigation patterns, page views and duration, and features used (video, audio, and document downloads). Implementation [I]. Fidelity and consistency of delivery will be measured. Maintenance [M] - Additional surveys will elicit perceptions of the potential sustainability of *iCare* from representative clinician and health service multi-disciplinary teams (*n* = 10), Government representatives (*n* = 5), consumers (*n* = 5) and community partners (*n* = 5).

### Ethical considerations

The study will be conducted according to Australia’s National Health and Medical Research Council’s National Statement on Ethical Conduct in Human Research 2007, updated 2018, and the World Medical Association Declaration of Helsinki 2013. All data collected from Qualtrics will be de-identified and stored in an SPSS spreadsheet. Identifiable participant information including name, phone number and email address will be stored in a password protected Microsoft Excel document, saved to Deakin University, Faculty of Health, School of Nursing secure drive. All electronic records will be stored on stored on a secure drive, accessible only via a password protected computer; only the project manager and project investigator will have access to the files. Only de-identified data will be shared with other members of the research team e.g., statistician and economic evaluation support. Any paper documents will be stored in a locked filing cabinet and only the project manager and project investigator will have access to the cabinet.

Seven years from the last date of publication, all electronic documents will be permanently deleted and paper files will be disposed of in confidential waste at Deakin University. Sharing of deidentified data will be available upon reasonable request by another research team within these 7 years.

### Auditing

This study may be audited by the Deakin University Human Research Ethics Committee.

### Protocol amendments

Any protocol amendments will be submitted and approved by the governing Human Research Ethics Committee, with relevant changes sent to the Australian and New Zealand Clinical Trial Registry.

## Discussion

With few advocacy groups and support systems for people with upper GI or HPB cancers, *iCare* is designed to address a significant gap in supportive care for people diagnosed with these cancers and their carers. *iCare* has been designed to improve health outcomes and reduce health care costs across the Australian community [42, 43]. This study will therefore deploy a clinical trial designed to provide feasibility and acceptability data which will provide the necessary information for consumers, advocacy and community organisation partners to scale the delivery of *iCare* to patients and their carers via stakeholder websites.

*iCare*’s potential for impact and translation will be supported by demonstrating significant improvement in the lives of people affected by upper GI or HPB cancers and their carers across the disease trajectory, through a practical and easy-to-use platform that could support people anytime and anywhere in Australia. We will monitor recruitment, and key deliverables, including uptake, data integrity and problem solving, program usage, tracking progress against the project milestones. Partnering with key national consumer and advocacy groups, consumers and government, will achieve critical knowledge-to-practice impact, and will inform the next stage of delivery. We anticipate the study will take approximately three years to complete. In our assessment, *iCare* represents a low-risk, high-value proposition. The team and partners have the required track record, skills and resources, and a solid research plan, governance structure and risk mitigation strategy.

We expect the study outcomes will inform the potential economic and social impact of upper GI cancers by reducing health system and societal costs as well as demonstrating how *iCare* can support people with significant needs and their carers across the community with evidence-based supportive care services being embedded in the community and accessible through our partner organizations.

## Data Availability

The datasets used and/or analysed during the current study will be available from the corresponding author on reasonable request.

## Abbreviations

AQoL4D: Assessment of Quality of Life – Four Dimensions
CQOLC: Caregiver Quality of Life Index-Cancer
FACT-G: Functional Assessment of Cancer Therapy – General
HPB cancers: Hepato-pancreato-biliary cancers
QALY: Quality Adjusted Life Years
QOL: Quality of Life
QR code: Quick Response code
RE-AIM Framework: Reach, Effectiveness, Adoption, Implementation, Maintenance Framework
RCT: Randomised Controlled Trial
SILS: Single Item Literacy Screener
SCNS: Supportive Cancer Needs Survey
SCNS-P&C: Supportive Cancer Needs Survey – Partners and Caregivers
T1: Time one follow-up point at baseline
T2: Time two follow-up point at 3 months post baseline
T3: Time three follow-up point at 6 months post baseline
UGICR: Upper Gastrointestinal Cancer Registry
Upper GI: Upper Gastrointestinal
URL: Uniform Resource Locator
ZBI-12: Zarit Burden Interview 12

## References

[1] Australian Institute of Health and Welfare. Health system expenditure on cancer... in Australia, 2015–16. 2021.

[2] Berman R (2020). Supportive Care: An Indispensable Component of Modern Oncology. Clin Oncol.

[3] Pancare. State of the Nation: Upper Gastrointestinal (GI) Cancers in Australia. Pancare; 2022.

[4] Australian Institute of Health and Welfare. Colorectal and other digestive-tract cancers. AIHW; 2018.

[5] Australian Institute of Health and Welfare (2022). Cancer in Australia 2021.

[6] Kissane DW (2004). Psychiatric disorder in women with early stage and advanced breast cancer: a comparative analysis. ANZ J Psychiatry.

[7] Mercadante S (2015). Sleep Disturbances in Patients With Advanced Cancer in Different Palliative Care Settings. J Pain Symptom Manage.

[8] Grabsch B (2006). Psychological morbidity and quality of life in women with advanced breast cancer: a cross-sectional survey. Palliat Support Care.

[9] Otutaha B, Srinivasa S, Koea J (2019). Patient information needs in upper gastrointestinal cancer: what patients and their families want to know. ANZ J Surg.

[10] Ugalde A (2021). Effective integration of caregivers and families. Aust J Gen Pract.

[11] Al-Janabi H, McCaffrey N, Ratcliffe J (2013). Carer preferences in economic evaluation and healthcare decision making. Patient.

[12] Olson RE (2012). Is cancer care dependant on informal carers?. Aust Health Rev.

[13] Glajchen M (2004). The emerging role and needs of family caregivers in cancer care. J Support Oncol.

[14] Cancer Council NSW (2007). Cost of Cancer in NSW.

[15] Livingston PM (2020). Outcomes of a randomized controlled trial assessing a smartphone Application to reduce unmet needs among people diagnosed with CancEr (ACE). Cancer Med.

[16] Dober, M., et al., Perspectives on an Acceptance and Commitment Therapy (ACT) based program for patients with inflammatory bowel disease and comorbid anxiety and/or depressive symptoms. Psychotherapy Research, 2020: p. 1–14. 10.1080/10503307.2020.1813915 32892715

[17] Ugalde A (2020). Priorities for cancer caregiver intervention research: A three-round modified Delphi study to inform priorities for participants, interventions, outcomes, and study design characteristics. Psychooncology.

[18] Chow, R., et al., Interventions to improve outcomes for caregivers of patients with advanced cancer: a meta-analysis. JNCI: Journal of the National Cancer Institute, 2023. 115(8): p. 896–908. 10.1093/jnci/djad075 PMC10407714 37279594

[19] Zhai S (2023). Digital health interventions to support family caregivers: An updated systematic review. Digit Health.

[20] Heynsbergh N (2018). Feasibility, useability and acceptability of technology-based interventions for informal cancer carers: a systematic review. BMC Cancer.

[21] Rosenbloom ST, Steitz BD, Warner JL (2018). Window of Opportunity: Patient Portals and Cancer. J Oncol Pract.

[22] Yazdanian A (2022). Mobile-Based Self-management Application Requirements for Patients With Gastric Cancer: Quantitative Descriptive Study of Specialist and Patient Perspectives. JMIR Cancer.

[23] Winter, N., et al., iCare – Protocol... in Int Psych-Onco Soc Conference. 2022.

[24] Darling G (2006). Validation of the functional assessment of cancer therapy esophageal cancer subscale. Cancer.

[25] Bonevski B (2000). Evaluation of an instrument to assess the needs of patients with cancer. Support Care Rev Group Cancer.

[26] Bedard M (2001). The Zarit Burden Interview: a new short version and screening version. Gerontologist.

[27] Girgis A, Lambert S, Lecathelinais C (2011). The supportive care needs survey for partners and caregivers of cancer survivors: development and psychometric evaluation. Psychooncology.

[28] Weitzner MA (1999). The Caregiver Quality of Life Index-Cancer (CQOLC) scale: development and validation of an instrument to measure quality of life of the family caregiver of patients with cancer. Qual Life Res.

[29] Glasgow RE, Vogt TM, Boles SM (1999). Evaluating the public health impact of health promotion interventions: the RE-AIM framework. Am J Public Health.

[30] Upper Gastrointestinal Cancer Registry. Who are we; our purpose; why participate. n.d 11.08.2023]; Available from: https://ugicr.org.au.

[31] Cancer Council Australia. 13 11 20. n.d 11.08.2023]; Available from: https://www.cancer.org.au/about-us/how-we-help/support/stories/support/131120.

[32] Heynsbergh N (2019). Development of a Smartphone App for Informal Carers of People With Cancer: Processes and Learnings. JMIR Form Res.

[33] Heynsbergh N (2019). A Smartphone App to Support Carers of People Living With Cancer: A Feasibility and Usability Study. JMIR Cancer.

[34] Morris NS (2006). The Single Item Literacy Screener: Evaluation of a brief instrument to identify limited reading ability. BMC Fam Pract.

[35] Short CE (2018). Measuring Engagement in eHealth and mHealth Behavior Change Interventions: Viewpoint of Methodologies. J Med Internet Res.

[36] Dionne-Odom JN (2022). A lay navigator-led, early palliative care intervention for African American and rural family caregivers of individuals with advanced cancer (Project Cornerstone): Results of a pilot randomized trial. Cancer.

[37] Hawthorne G, Richardson J, Osborne R (1999). The Assessment of Quality of Life (AQoL) instrument: a psychometric measure of health-related quality of life. Qual Life Res.

[38] Richards-Jones S (2019). An economic evaluation of a telephone outcall intervention for informal carers of cancer patients in Australia: An assessment of costs and quality-adjusted-life-years. Psychooncology.

[39] Heckel L, Gunn KM, Livingston PM (2018). The challenges of recruiting cancer patient/caregiver dyads: informing randomized controlled trials. BMC Med Res Methodol.

[40] Luckett T (2010). Assessing HRQOL in gynecologic oncology: a systematic review. Int J Gyn Canc.

[41] Preston NJ (2013). Recommendations for managing missing data, attrition and response shift in palliative and end-of-life care research: part of the MORECare research method guidance on statistical issues. Palliat Med.

[42] Jefford M (2022). Improved models of care for cancer survivors. Lancet.

[43] Emery J (2022). Management of common clinical problems experienced by survivors of cancer. The Lancet.

